# Supplementary material for: The architects of bacterial DNA bridges: a structurally and functionally conserved family of proteins
Source: Open Biol. 2019 Dec 4;9(12):190223. doi: 10.1098/rsob.190223 (PMC6936261; doi:10.1098/rsob.190223)
Supplement: Electrostatics and fold topology of H-NS-like proteins [file rsob190223supp1.pdf]

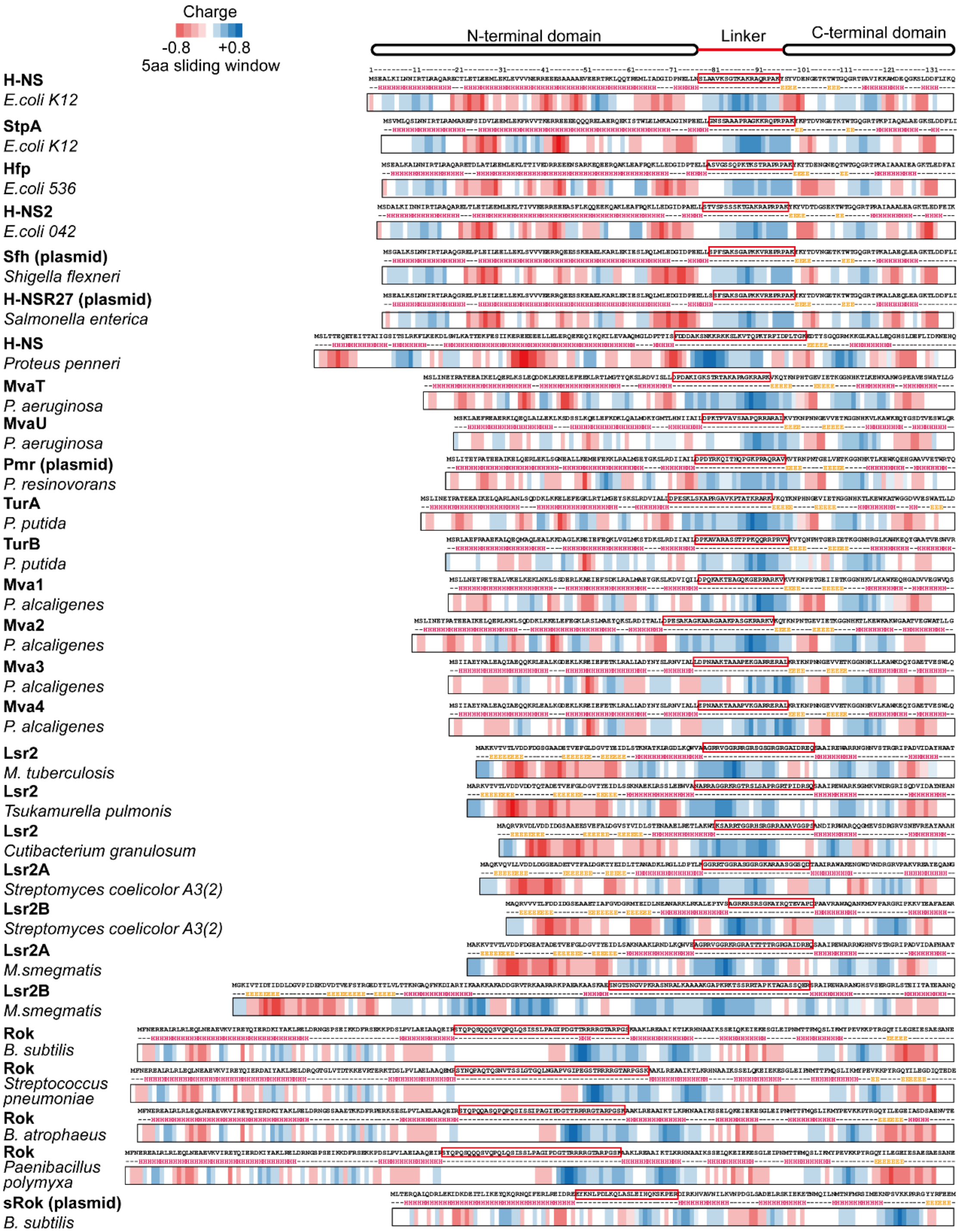

Supplementary figure: Electrostatics and fold topology of H-NS-like proteins. Comparison of the charge distribution of the four families of H-NS like protein across a range of species. The average charge of a five-amino acid window as analyzed with EMBOSS charge. Positively and negatively charged regions are colored blue and red respectively. Unknown secondary structures were predicted using JPRED(49). α-

Helices are represented with (H) in pink, β-sheets with (E) in yellow and random coils with (-) in black. The linker domain is indicated with a red box. H-NS, *E. coli* strain K-12, NP_415753.1; StpA*, E. coli* strain K-12, NP_417155.1; Hfp, *E. coli* strain 536, ABG69928.1; H-NS2, *E. coli* strain 042, CBG35667.1; Sfh, *Shigella flexneri 2a*, AAN38840.1; H-NSR27, *Salmonella enterica subsp. enterica serovar Typhi* strain CT18, NP_569380.1; H-NS, *Proteus penneri*, SUB98598.1; MvaT, *P. aeruginosa* PAO1, NP_253005.1; MvaU, *P. aeruginosa* CLJ1, PTC37345.1; Pmr, *P. resinovorans*, NP_758612.1; TurA, *P. putida*, SUD72464.1; TurB, *P. putida*, VEE40761.1; Mva1/2/3/4_PALC, *P. alcaligenes* RU36E SIQ98833.1, SIQ72658.1, SIP93681.1 and SIP94365.1; Lsr2_MTUB, *M. tuberculosis* H37Rv, NP_218114.1; Lsr2, *Tsukamurella pulmonis*, SUP14481.1; Lsr2, *Cutibacterium granulosum*, SNV28945.1; Lsr2A/B_SCOEL, *S. coelicolor* A(3)2 , CAB40875.1 and CAB56356.1; Lsr2A/B_MSMEG, *M. smegmatis* MKD8, AWT56911.1 and AWT52048.1; Rok, *B. subtilis* strain 168, NP_389307.1; Rok, *Streptococcus pneumonia*, CVM76913.1; Rok, *B. atrophaeus*, KFK83781.1; Rok, *Paenibacillus polymyxa*, SPY12450.1; sRok, *B. subtilis subsp. natto*, YP_004243533.1.
